# A unique resistance mechanism is associated with *RBgh2* barley powdery mildew adult plant resistance

**DOI:** 10.1007/s00122-023-04392-0

**Published:** 2023-05-30

**Authors:** Paula Moolhuijzen, Cynthia Ge, Elzette Palmiero, Simon R. Ellwood

**Affiliations:** Centre for Crop and Disease Management, School of Molecular and Life Sciences, Curtin University, Bentley, WA 6102 Australia

## Abstract

**Key message:**

Gene expression at the *RBgh2* locus indicates involvement in cAMP/G-protein-coupled signalling and innate immunity in barley powdery mildew adult plant resistance.

**Abstract:**

Barley powdery mildew is a globally significant disease, responsible for reduced grain yield and quality. A major effect adult plant resistance gene, *RBgh2*, was previously found in a landrace from Azerbaijan. The atypical phenotype suggested different underlying genetic factors compared to conventional resistance genes and to investigate this, genome-wide gene expression was compared between sets of heterogeneous doubled haploids. *RBgh2* resistance is recessive and induces both temporary genome-wide gene expression changes during powdery mildew infection together with constitutive changes, principally at the *RBgh2* locus. Defence-related genes significantly induced included homologues of genes associated with innate immunity and pathogen recognition. Intriguingly, *RBgh2* resistance does not appear to be dependent on salicylic acid signalling, a key pathway in plant resistance to biotrophs. Constitutive co-expression of resistance gene homologues was evident at the 7HS *RBgh2* locus, while no expression was evident for a 6-transmembrane gene, predicted *in silico* to contain both G-protein- and calmodulin-binding domains. The gene was disrupted at the 5′ end, and G-protein-binding activity was suppressed. *RBgh2* appears to operate through a unique mechanism that co-opts elements of innate immunity.

**Supplementary Information:**

The online version contains supplementary material available at 10.1007/s00122-023-04392-0.

## Introduction

Barley powdery mildew is caused by the airborne ascomycete fungus *Blumeria graminis* f. sp. *hordei* (*Bgh*). Two forms of genetic resistance predominate in barley cultivars; pathogen race-specific major resistance genes that are prone to breaking down, and broad-spectrum durable resistance achieved through the use of recessive alleles of the *mlo* gene (Jørgensen 1992; Reinstädler et al. 2010). *mlo* resistance has remained effective for over 50 years; however, *mlo* alleles are associated with pleiotropic effects and are implicated in increased susceptibility to several facultative fungal pathogens (Jarosch et al. 1999; Kumar et al. 2001; McGrann et al. 2014). Natural variants such as *mlo-11* (*cnv2*) show reduced necrotic leaf lesions compared to other *mlo* alleles (Ge et al. 2016, 2021); nonetheless, extensive use of *mlo* in modern cultivars exerts strong adaptive pressure on the pathogen.

An alternative category of resistance is adult plant resistance (APR), which may be considered as non-race-specific and potentially durable (Hwang and Heitefuss 1982). APR resistance genes do not provide absolute resistance as conferred by major R-genes but different levels of partial resistance. APR against mildew has been reported in both wheat and barley although the underlying genetic defence mechanisms remain largely unresolved. In wheat, APR may be single or polygenic (Asad et al. 2014; Burdon et al. 2014; Johnson et al. 2003) and may provide resistance to more than one fungal pathogen, for example *Lr34/Yr18/Pm38* and *Lr67/Yr46/Sr55/Pm46* in wheat (Herrera-Foessel et al. 2014; Krattinger et al. 2009).

Ge et al. (2021) defined a barley APR locus, *RBgh2*, in an Azerbaijan landrace, Eth069. The resistance exhibited infrequent epidermal cell hypersensitive responses, normally indicative of race-specific resistance, and was characterised by penetration resistance associated with cell wall appositions and large 3,3-diaminobenzidine (DAB) halos with diffuse cytosolic vesicle‐like bodies. In adult leaves, small sparse mildew pustules were observed which is characteristic of APR whereby disease progression is limited. We hypothesised that these features suggested different underlying host genetic responses compared to classical race-specific or *mlo* resistance (Ge et al. 2020). Since the dominance of *RBgh2* was not known, we set out to establish this, and, to investigate pathways and genes underlying *RBgh2*-mediated APR, gene expression involving sets of doubled haploids (DH) lines was studied to minimise genotype-specific expression changes in heterogeneous backgrounds unrelated to pathogen response.

## Materials and methods

### Barley and *Bgh* materials and propagation

Doubled haploid lines used in this study were produced from a cross between landrace Eth069 (PI 68192) and an Australian malting cultivar (cv.) Baudin as described in Ge et al. (2021). *RBgh2* was mapped to a 0.69 centiMorgans (cM) interval, based on a genetic map containing 966 genotyping-by-sequencing codominant SNP markers with an average marker spacing of 1.16 cM. Four lines containing the *RBgh2* APR gene (Ba69_012, Ba69_109, Ba69_288 and Ba69_304) and four susceptible lines (Ba69_106, ba69_118, Ba69_134 and Ba69_582) were selected for gene expression analysis, based on their disease phenotype and the SNP genotypic composition. Over 99% of each parental genome was shared across the lines in each category, with the remainder composed mainly of a region containing a second Eth069 APR locus, *RBgh3*, excluded on the basis of genetic markers indicating the susceptible parent genotype. Western Australian *Bgh* isolate Chi-001 was cultured for inoculations on detached leaves in benzimidazole agar plates (Tucker et al. 2013).

### F_1_ progeny disease phenotyping

The dominance of *RBgh2* was determined in crosses between the susceptible cv. Baudin and the resistant DH line *RBgh2* 12. To verify heterozygous F_1_ individuals, SNPs were detected by Sanger sequencing of HORVU7Hr1G002390. A CAPS marker was selected at the 3′ end, with PCR product cleavage by the restriction enzyme *Mse*I. The PCR primers (5′–3′) used were CTTGCATCATGGGAAAACCAAC and GTTAACATGCTATGATTAAAAGTGTGC. F_1_ plants were grown to the fifth leaf stage and evaluated at 14 dpi following inoculation with *Bgh* isolate Chi-001.

### Plant growth and inoculation for RNA extraction

Barley lines were sown in 20-cm diameter pots containing UWA Bio Mix (Richgro Garden Products, Jandakot, WA) and grown under a 12-h day–night cycle at 22 °C and 18 °C, respectively, in a climate-controlled growth room (Conviron Asia Pacific Pty Ltd, VIC, Australia). Four replicates of each DH line were planted for *Bgh*-inoculated and non-inoculated control samples, together with cv. Baudin as a standard susceptible control. Plants were placed in a randomised complete block design for each treatment and grown until the sixth leaf was fully expanded at around 5½ weeks post-germination, as APR is growth stage dependent and is fully effective from the fifth leaf (Ge et al. 2021).

Whole plant inoculations were conducted by first marking the borders of eight-centimetre 6th leaf sections with Micropore surgical tape (3 M, Saint Paul, MN, USA). The target sections were inoculated by gently tapping infected detached barley leaves from a height of 15 cm. Thereafter, entire plants were inoculated from a height of 50 cm. Leaf sections were harvested at 48-h post-inoculation (hpi) and flash frozen in liquid nitrogen. Susceptible DH lines and cv. Baudin were inspected at 7 dpi to confirm successful infection.

### RNA extraction and sequencing

RNA was extracted with a RNeasy Plant Mini Kit (Qiagen, Hilden, Germany) following the manufacturer’s protocol. Sample aliquots were taken to check RNA integrity on an agarose gel, the concentration measured with a Nanodrop 2000 (Thermo Fisher Scientific Inc., Wilmington, DE, USA), then samples stored at − 80 °C. Before shipment, RNA samples were thawed on ice and 1 µg of each transferred to GenTegra-RNA tubes (Pleasanton, CA, USA) and freeze-dried for 1½ h. RNA samples were sent to Novogene (Singapore) for directional mRNA library preparation followed by 150 bp (base pairs) paired-end read sequencing on a NovaSeq 6000 platform (Illumina Inc., San Diego, CA, USA).

### RNA sequence analysis

The IBSC Morex barley reference genome assembly (European Nucleotide Archive accession GCA_901482405.1) was downloaded from EnsemblPlants (Howe et al. 2020) with low complexity repeats soft masked and gene annotation v48. The genome and gene annotation was indexed using Star v2.7 (Dobin et al. 2013) with the options—sjdbGTFfeatureExon CDS—sjdbOverhang 149.

The paired-end reads were trimmed for adapters and random 5′ hexamer primers using Trimmomatic v0.39 (Bolger et al. 2014) with the following options (HEADCROP:12 ILLUMINACLIP:ADAP:2:30:5 LEADING:10 TRAILING:10 SLIDINGWINDOW:4:25 MINLEN:50). Trimmed reads were aligned to the indexed barley genome using the STAR RNA aligner v2.7 (—twopassMode Basic—quantMode GeneCounts—outSAMtype BAM SortedByCoordinate). Sample metadata and a gene expression matrix were analysed using R v4.0.3 and the R package DESeq2 v1.30.0 (Love et al. 2014) for differential gene expression analysis based on negative binomial (a.k.a. Gamma-Poisson) distribution. Differentially expressed genes (DGEs) were significant at an adjusted *p *value (false discovery rate) ≤ 0.05 and log_2_ fold change ≥ 1. The analyses and expression data are available in RStudio v1.3.1093 (RStudio-Team 2020) markdown script at https://github.com/ccdmb/barley-DH-pow-rnaseq. Figure plots were constructed using R v4.0.3 and the packages chromPlot v1.10.0 and ggplot2 v3.3.3. *De novo* sample transcript assembly was conducted using Spades v3.13.0 (Bankevich et al. 2012) (–rna -m 63 -t 24), clustered using CD-HIT v4.6.8 (Fu et al. 2012; Li and Godzik 2006) (-T 10 -c 0.98) and coding sequence predicted using CodAn v1.2 (Nachtigall et al. 2021) (-c6 -H 98). Gene expression data from a previous study of powdery mildew infection of barley near-isogenic *mlo* lines (NILs) (Ge et al. 2020) were used to compare expression around the 7H *RBgh2* locus and are also available through the GitHub link above.

### Gene ontology term analysis of differentially expressed genes

Functional enrichment for differentially expressed genes was determined using R version 4.0.3, topGO v2.42.0 (Alexa et al. 2006) and Rgraphviz v2.34.0 (Hansen et al. 2020). The gene ontology (GO) terms for significantly differentially expressed genes (SDEGs) were compared to the ontologies for all genes in the barley genome (population background) to determine enrichment using Fisher’s exact test with *p *values ≤ 0.01. The analyses and data are available in RStudio v1.3.1093 (RStudio-Team 2020) markdown notebook https://github.com/ccdmb/barley-DH-pow-rnaseq.

### Protein tertiary structure predictions

Protein tertiary structural predictions were conducted using ColabFold (Mirdita et al. 2021), powered by AlphaFold2 (Jumper et al. 2021) and RoseTTAFold (Baek et al. 2021) with default settings (MMseqs2 multiple sequence alignment, homo-oligomer 1, subsample true, number relax 0, use turbo true, rank by pLDDT, number of models 5, number of samples 1, max recycles 3 and tol 0). AlphaFold-Multimer was used for the prediction of the protein complexes between multiple proteins (Evans et al. 2021).

### PCR amplification of Morex HORVU7Hr1G002390

PCR primers were designed to overlapping regions of the Morex HORVU7Hr1G002390 gene model (HORVU.MOREX.r3.7HG0636850), including approximately 1000 bp of the 5′ and 3′ UTRs (Table S1). PCR products were visualised by electrophoresis on a 1% TAE agarose gel.

## Results

### *RBgh2* resistance is recessive

**Fig. 1:**
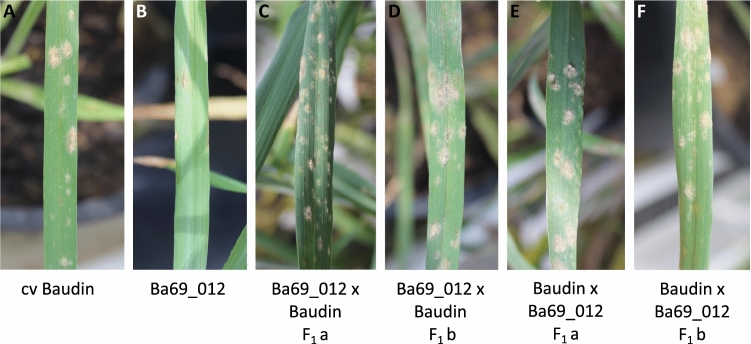
Powdery mildew disease phenotypes of *RBgh2* F_1_ progeny. Symptoms are shown for F_1_ individuals from crosses between cv. Baudin and the *RBgh2-*containing DH line Ba69_012 at 14 dpi. Parental lines are shown on the left (panels **a** and **b**) and F_1_ individuals in panels **c**–**f**

To determine dominance of *RBgh2*, a DH line was selected to generate F_1_ progeny as the original donor parent, Eth069, was found to be difficult to cross (Ge et al. 2021). Reciprocal crosses were made between the powdery mildew-susceptible parent cv. Baudin and DH line 12. All F_1_ progeny were susceptible, indicating that *RBgh2* is recessive and not influenced by mitochondrial or chloroplast genes (Fig. 1).

### RNA read coverage and summary of SDEGs in doubled haploid leaf samples

RNA sequencing (RNA-seq) yielded over 20 million reads for each replicate sample, except sample AU1_C, which yielded over 18 million (Figure S1). On average, 92% of total sample sequences mapped to the cv. Morex barley reference genome, with 83% of read alignments mapping uniquely and 9% to multiple loci. Some 24,346 barley genes (56% of all genes) were expressed with a read count ≥ 30. A principal component analysis (PCA) showed genotype accounted for 13% of the variation between samples while treatment (*Bgh*-inoculated or non-inoculated controls) accounted for 39% of variation (Figure S2).

**Fig. 2:**
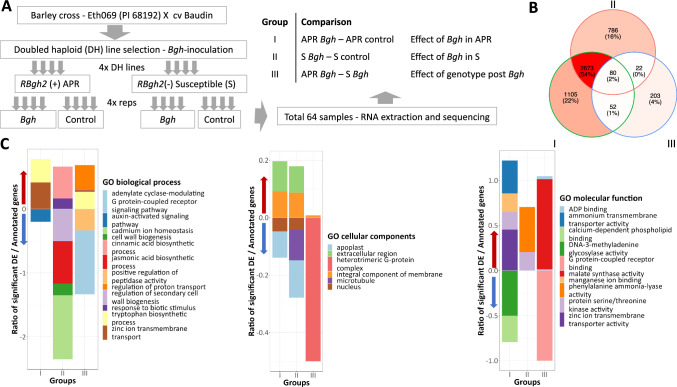
Gene expression and GO term differences between *RBgh2* barley powdery mildew-resistant and -susceptible DH lines. **a** An overview of the RNA experimental design and grouped sample comparisons. **b** Venn diagram of significantly differentially expressed genes (SDEGs). Groups I and II represent *Bgh*-inoculated APR and susceptible lines relative to their respective control samples, and group III represents APR *Bgh-*inoculated relative to susceptible *Bgh-*inoculated samples. **c** Plots for significant GO term enrichments for SDEGs. Biological processes, cellular components and molecular functions are shown from left to right. The *y*-axis displays the ratio of significantly DEGs/total number of genes annotated for a GO term (in the barley genome) for each group comparison (*x*-axis), with the same comparison groups as in **b**. GO terms represented by induced genes have positive values on the *y*-axis and suppressed genes by negative values on the *y*-axis

Among significantly differentially expressed genes (SDEGs) between different treatments, a PCA showed four distinct clusters with 38% and 17% of the variation explained by treatment and genotype, respectively (Figure S3). In inoculated APR DH lines (group I, Fig. 2a), a total of 3910 SDEGs were identified compared to controls (Fig. 2b), with the majority, 2430, induced (Table S2a). Among inoculated susceptible DH lines (group II), 3561 SDEGs were identified compared with the controls, and of these 2280 genes were induced (Table S2b). Overall, a total of 2753 DE genes were shared between inoculated APR (group I) and susceptible (group II) genotypes compared to their respective controls (Fig. 2b).

### GO terms enriched by SDEGs in APR and susceptible genotypes following *Bgh* inoculation

The APR DH SDEGs were significantly enriched for 44 GO term molecular functions and 37 biological processes, and the susceptible DH SDEGs were significantly enriched for 43 molecular functions and 46 biological processes, both compared to non-inoculated controls (Table S3). In each comparison, protein serine/threonine kinase activity was the most significant molecular function (*p *values of 4.5e^−29^ and 1e^−30^).

Major GO term differences between the APR lines during infection as compared to the controls were for increased manganese ion-binding, zinc ion and ammonium transmembrane transporter activities and tryptophan biosynthesis. Calcium-dependent phospholipid binding was supressed, together with DNA-3-methyladenine glycosylase activity and auxin signalling. In susceptible lines compared to their controls, terms for phenyl ammonia lyase (PAL) and cinnamic acid biosynthesis increased, while cadmium ion homeostasis, JA activity and several processes with GO terms associated with cell wall biogenesis were reduced.

In comparisons between SDEGs in the APR and susceptible DH lines following powdery mildew inoculation only, a modest number of genes (357) were differentially expressed (Table S2c). These were significantly enriched for 18 GO term molecular functions and 13 biological processes (Table S3), with ADP binding the most significant molecular function (*p *value of 7e−07). A total of 234 SDEGs were induced in the APR DHs relative to the susceptible DHs with enriched GO terms involved in ADP binding, tryptophan biosynthesis, regulation of proton transport, response to biotic stimulus and protein serine/threonine kinase activity (Fig. 2c). In the tryptophan biosynthetic pathway, four of the eight homologues for five key enzymes (trpA, trpB, trpC, trpD and trpG) were significantly induced (Figure S4). In contrast, enriched GO terms for supressed SDEGs included the adenylate cyclase-modulating G-protein-coupled receptor signalling pathway (including G-protein-coupled receptor binding) and positive regulation of peptidase activity, while malate synthase was prominent among GO terms for induced SDEGs in APR lines as compared to susceptible lines (Fig. 2c).

### Defence-related genes differentially expressed during powdery mildew infection of *RBgh2* DH lines

A large proportion (142/357) of SDEGs identified in inoculated APR *RBgh2* DH lines compared to susceptible DH lines were involved in pathogen sensing, signalling and pathogen defence responses (Table 1). Of these, over 80% were annotated as having the pathogen recognition functions of receptor-like kinase, NB-ARC-like, LRR domain-containing and calmodulin-binding genes. Induction on genes in the SA pathway, which is commonly associated with resistance to biotrophic fungal pathogens in plants, was not observed in the APR lines. In contrast, two genes involved with JA and ethylene signalling were supressed. The majority of transcription factors (7/9) were also supressed in the APR lines.

**Table 1:** Overview of barley defence-related genes with significant expression changes in APR as compared to susceptible DH lines during *Bgh* infection

Defence categories	Gene or annotation identifier	APR v S during PM infection^a^	
		Induced	Suppressed
*Pathogen sensing*			
Receptor-like kinases	IPR011009	21	8
NB-ARC	IPR002182	1	0
NB-ARC LRR	IPR002182, IPR032675	12	3
LRR	IPR032675	18	8
MLO-like	IPR004326	1^b^	1^b^
Calmodulin binding	GO:000551	33	9
*Signalling*			
Transcription factors	GO:0006355	2	7
Jasmonate pathway: ZIM-domain (JAZ) repressor of Jasmonate (JA) signalling	HORVU7Hr1G041260	0	1
Ethylene pathway: ACC synthase	HORVU4Hr1G009800	0	1
*Oxidative degradation*			
Cytochrome P450s	IPR001128	6	3
*Biosynthesis*			
Tryptophan	HORVU1Hr1G093480, HORVU4Hr1G061120, HORVU4Hr1G083210, HORVU7Hr1G114660	4	0
Serotonin (via tryptamine; Tryptophan decarboxylase)	HORVU2Hr1G114390, HORVU2Hr1G114450	2	0
*Pathogenesis related*			
PR5 Thaumatin-like	IPR001938 HORVU5Hr1G005180	1	0
PR7 Subtilisin-like	IPR000209 HORVU3Hr1G077950	1	0
Sugar transporter-like	IPR005828	2	0

^a^Number of significantly differentially expressed barley genes in APR DH lines compared to susceptible DH lines, based on adjusted *p *value ≤ 0.01 and absolute log2 FC> 1

^b^Note IPR004326 is also represented in the calmodulin-binding category

Among biosynthetic and catabolic genes, six genes in the tryptophan pathway were exclusively expressed in the APR lines and were annotated as having roles in tryptophan biosynthesis, decarboxylation and possibly serotonin production. Cytochrome P450 genes were expressed in both genotypes although six were induced and three suppressed in the APR lines compared to the susceptible lines. Other genes notably induced in APR lines included pathogenesis-related (PR) thaumatin-like *PR5* (HORVU5Hr1G005180) and subtilisin-like *PR*7 (HORVU3Hr1G077950).

### Gene expression at the 7H *RBgh2* locus

**Fig. 3:**
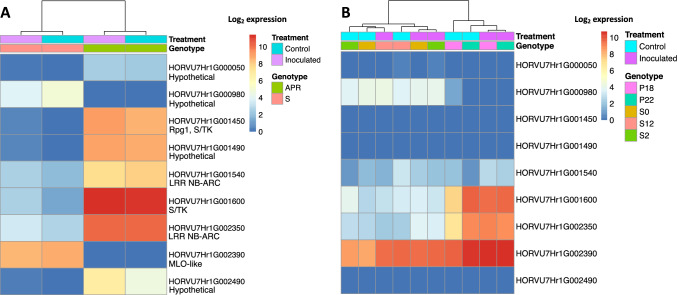
Graphical representation of gene expression around the *RBgh2* locus. Average normalised expression is shown for genes within approximately 2 Mbp of closest *RBgh2* genetic marker, based on an adjusted *p* value ≤ 0.05 and absolute log_2_ FC> 5. **a** Doubled haploid (DH) line gene expression. *Bgh*-inoculated samples are indicated in purple and controls in blue. APR *RBgh2* DH lines are shown in green and susceptible lines in red. **b** Gene expression in *mlo* near-isogenic lines (NILs) from Ge et al. (2020). NILs are S2 (*mlo-11*(*cnv2*), in green), S12 (*mlo-11*, in red) and P22 (*mlo-5*, in cyan). Recurrent parent control lines are S0 (cv. Baudin, for *mlo-11*(*cnv2*) and *mlo-11*, in tan) and P18 (cv. Pallas, for *mlo-5*, in light purple) (colour figure online)

*RBgh2* DH lines were unique in showing constitutive expression of several genes in the distal region of chromosome 7HS under the experimental conditions (Fig. 3a and Figure S5). These co-locate to within approximately 2 Mb of the closest genetic marker for the *RBgh2* locus at 2,474,271 bps (Ge et al. 2021). Highly expressed genes (absolute log_2_ fold change ≥ 5, Table 2) were represented by three protein kinases, HORVU7Hr1G001600, HORVU7Hr1G001450 and HORVU7Hr1G001490, together with a NB-ARC LRR-like resistance gene (HORVU7Hr1G002350). These genes showed little or no expression in the susceptible DH lines, but were expressed in both the APR *Bgh*-inoculated and control samples. A second NB-ARC LRR gene (HORVU7Hr1G001540) and a hypothetical disease resistance protein (HORVU7Hr1G000050) were also constitutively expressed at a moderate level. A hypothetical aspartic peptidase domain-containing protein integral to the membrane (HORVU7Hr1G002490) was also highly expressed (log_2_ FC 9.87) (Table 2).

**Table 2:** Highly regulated barley genes on chromosome 7H within approximately 2 Mb of the closest *RBgh2* genetic marker

Gene/marker	Mean	Log2 FC	Adj. *p *value	Gene name (Ensembl)	InterPro/GO/PANTHER domain descriptions	Chr:7H location (bp)
Barley marker eth9	–	–		–	–	2,474,271
HORVU7Hr1G000050	2.72	5.17	1.17E−11	–	Disease resistance protein	50,769–51,791
HORVU7Hr1G000980	13.94	− 6.67	2.75E−27	–	Fatty acid hydroxylase, endoplasmic reticulum membrane	2,100,992–2,105,040
HORVU7Hr1G001450	239.50	11.62	1.06E−107	Rpg1, stem rust resistance protein	Protein kinase domain; serine/threonine protein kinase active site	3,257,424–3,268,163
HORVU7Hr1G001490	228.60	11.44	8.76E−105	–	Protein kinase-like domain superfamily	3,329,219–3,330,517
HORVU7Hr1G001520	4.53	5.52	6.08E−09	–	–	3,379,276–3,382,077
HORVU7Hr1G001540	118.35	5.32	7.90E−55	–	NB-ARC; P-loop-containing nucleoside triphosphate hydrolase; leucine-rich repeat domain superfamily; Rx, N-terminal	3,389,278–3,396,099
HORVU7Hr1G001600	1283.67	9.41	0	ABC1037, serine/threonine kinase-like	Protein kinase domain (Pfam:PF18052); protein kinase-like domain superfamily; ATP-binding site; serine/threonine protein kinase active site	3,444,741–3,467,152
HORVU7Hr1G002350	603.46	7.17	0	–	NB-ARC; P-loop-containing nucleoside triphosphate hydrolase; leucine-rich repeat domain superfamily	4,471,843–4,480,358
HORVU7Hr1G002390	317.41	− 12	1.04E−77	MLO-like protein	Mlo-related protein, integral component of membrane, calmodulin binding, defence response	4,608,055–4,612,791
HORVU7Hr1G002490	52.33	9.87	7.35E−38	–	Aspartic peptidase, xylanase inhibitor, integral component of membrane	4,763,442–4,764,158

Data are shown for DH APR lines at 48 hpi with *Bgh* (based on adjusted *p *value ≤ 0.01 and absolute log_2_ FC> 5)

No expression in APR lines at the locus was found for the gene (HORVU7Hr1G002390). To investigate the gene, eight primer pairs were designed encompassing the entire 4-kb coding region. In cv. Baudin, all primer pairs amplified PCR products but in Eth069, only primers targeting the second half of the gene (exons 10–12) were successful, indicating substantial gene disruption (Table S1). Two other genes exhibited repressed expression, a hypothetical fatty acid hydroxylase (HORVU7Hr1G000980) (Table 2) and a LRR domain-containing protein (HORVU7Hr1G002910), which had low expression (log_2_ FC 2) in susceptible DH lines (Figure S6).

### Gene expression at 7H *RBgh2* locus does not correspond with *mlo* resistance

To explore any potential relationship between *RBgh2* and *mlo* resistance, differentially expressed genes around the 7H *RBgh2* locus were also compared in pre-existing near-isogenic line (NIL) data (Ge et al. 2020). The NILs contain the *Mlo* alleles *mlo-11*(*cnv2*) and *mlo-*11 in a cv. Baudin background, with *mlo-5* in a cv. Pallas background. Relative *mlo* allele functional/phenotypic strengths are *mlo-11*(*cnv2*) < *mlo-11* < *mlo-5*, respectively. Negligible to no NIL gene expression was identified for the hypothetical genes HORVU7Hr1G000050 and HORVU7Hr1G002490, the protein kinase *Rpg1* (HORVU7Hr1G001450) and hypothetical kinase HORVU7Hr1G001490 (Fig. 3b). Furthermore, gene expression for the LRR NB-ARC domain-containing protein HORVU7Hr1G002350 and the serine/threonine protein kinase HORVU7Hr1G001600 was enhanced in the Pallas compared to the Baudin NILs. The expression patterns of these two genes together with HORVU7Hr1G000980 were similar to that of the APR DH lines suggesting a genotype-specific effect.

The *Mlo* paralogue HORVU7Hr1G002390 was expressed in all Baudin and Pallas NILs and for wild-type (WT) *Mlo* in cv. Baudin (S0) and cv. Pallas (P18). However, there was a higher expression in NILs with a Pallas genetic background and a slightly greater expression for inoculated samples dependent on the *mlo* allele. In ascending order of expression levels, lowest expression (log_2_ 8) was in non-inoculated controls for WT *Mlo* (S0) and *mlo-11*(*cnv2*) (S2). Medium expression (log_2_ 9) expression was in *Bgh*-inoculated cv. Baudin (S0), *mlo-11*(*cnv2*) (S2) and *mlo-*11 (S12), together with S12 (*mlo-*11) and cv. Pallas (P18) non-inoculated controls. Highest (log_2_ 10) expression was in *Bgh*-inoculated cv. Pallas (P18), inoculated *mlo-5* (P22) and *mlo-5* controls. Overall, the data suggest a different gene expression pattern among genes at the *RBgh2* locus in *mlo* lines, with HORVU7Hr1G002390 induced following *Bgh* infection which was dependent on both the genetic background and *mlo* allele.

### Homology of HORVU7Hr1G002390 with *Mlo*

HORVU7Hr1G002390 is classed at EBI as a paralogue of *Mlo* (HORVU.MOREX.r3.4HG0410620), the suppressor of broad-spectrum powdery mildew resistance (Piffanelli et al. 2002). To investigate the similarity, a full-length splicing isoform in cv. Baudin, tr_12967 was identified by *de novo* assembly of RNA reads (Figure S7). An alignment of predicted proteins between tr_12967 and HORVU7Hr1G002390 with *Mlo* showed a low identity of 32.5% (Figure S8). However, the C-terminal calmodulin (CaM)-binding domain was conserved for three of the five predicted hydrophobic residues important for CaM binding (Kim et al. 2002a).

### Constitutively expressed and repressed SDEGs outside the 7H *RBgh2* locus

Several genes in *RBgh2* APR DH lines were found in both inoculated and non-inoculated controls. These included two co-suppressed acyl-coenzyme A oxidases on chromosome 5H (HORVU5Hr1G006930) and 6H (HORVU6Hr1G020600) (Figure S6). On chromosome 7H, a WD40 repeat-containing protein (HORVU7Hr1G006140) and a hypothetical protein (HORVU7Hr1G006780) were also suppressed. Constitutively expressed genes were limited to a hypothetical protein (HORVU2Hr1G116490) on chromosome 2H. Genotype-dependent expression was also evident, for example five genes on chromosome 2 were expressed in all APR DH lines except Ba69_109, suggesting these are dispensable for resistance (Figure S6).

## Discussion

Gene expression changes governed by *RBgh2* primarily involve sensing and initiating defence responses, with conspicuous constitutive expression at the *RBgh2* locus. A prominent feature of *RBgh2* lines was a complete lack of expression of HORVU7Hr1G002390 and suppression of genes potentially involved in cAMP/G-protein-coupled signalling, with the implications explored below.

Genome-wide, resistance gene homologues (RLKs, LRRs and NB-ARCs) and a relatively large number of calmodulin-binding genes (33) were induced. Calcium ion influxes into the cytosol following recognition of either PAMPs (pathogen-associated molecular patterns), and/or DAMPs (damage-associated molecular patterns), play a pivotal role in the strength and specificity of plant immune responses through calmodulin-like proteins (Cheval et al. 2013; Seong and Matzinger 2004). The number of induced calmodulin-binding genes may be significant to *RBgh2* resistance given their participation in both pathotype-specific and innate immunity. In concert, suppression of calcium-dependent phospholipid binding was evident, which may mobilise cytosolic Ca^2+^ to activate defence responses (Lecourieux et al. 2006).

Key metabolic processes defining APR were the activation of cytochrome P450 monooxygenases and genes involving tryptophan biosynthesis, decarboxylation and synthesis of serotonin. P450 monooxygenases have diverse roles in biosynthetic and catabolic pathways that include the biosynthesis of core components of the phenylpropanoid pathway, leading to defence-related compounds such as cyanogenic glucosides and lignins (Schuler and Werck-Reichhart 2003). Their involvement in tryptophan metabolism has been demonstrated in *Arabidopsis*, where conversion of tryptophan to indole-3-acetaldoxime leads to the production of the Brassicaceae antimicrobial compounds camalexin and indole glucosinolate (Hull et al. 2000; Tzin and Galili). Subsequently, tryptophan-derived phytochemicals were shown to be essential for non-adapted or innate antifungal immunity (Consonni et al. 2010; Hiruma et al. 2013). In rice, serotonin, a derivative of decarboxylation of tryptophan to tryptamine, supresses growth of *Bipolaris oryzae* (Ishihara et al. 2008). Significant induction of malate synthase was also observed. Malate is involved in central metabolism and in plant defence provides NADPH reducing energy and backbone structures for defence compounds via the phenylpropanoid pathway (Casati et al. 1999).

Only two transcription factors were significantly induced in inoculated APR DH lines compared to susceptible lines. HORVU5Hr1G019000 has an FHY3/FAR1 (IPR031052) domain found in the *Arabidopsis* light signalling FHY3 and FAR1 proteins. Double null mutant studies showed *FHY3* and *FAR1* modulate plant immunity by negatively regulating ROS accumulation, oxidative stress and SA-dependent cell death (Ma et al. 2016) through the NB-LRR-mediated SA signalling pathway, reducing pathogenesis-related (*PR*) gene expression and resistance to *Pseudomonas syringae* bacteria (Wang et al. 2016). However, *PR* gene expression was unaffected in single mutants, and the relevance of the barley homologue requires further study. The second gene induced was a MADS-box transcription factor (HORVU5Hr1G082120). Such genes are key components of gene regulatory networks in response to stressful conditions, including oxidative stress (Castelan-Munoz et al. 2019).

Among *PR* genes, only HORVU5Hr1G005180, a *PR5* thaumatin-like orthologue and HORVU3Hr1G077950, a *PR7* subtilisin-like serine proteinase, were induced in *Bgh*-inoculated APR DH lines. Many thaumatin-like proteins have antimicrobial activities, where membrane permeability changes cause leakage of fungal hyphae cytoplasmic material, resulting in eventual cell rupture and an increased uptake of antifungal proteins (Sudisha et al. 2012). In *Arabidopsis*, *PR7*-like *AtSBT3.3* primes innate immune responses via chromatin remodelling of SA-dependent defence genes and MAP kinase activation (Gil et al. 2005).

### De-repression of defence-related genes

Surprisingly, only two genes involving the primary plant defence signalling pathways for salicylic acid (SA), jasmonic acid (JA) and ethylene (ET) showed significantly altered expression, both being supressed. These compounds have different roles against fungal pathogens depending on their modes of obtaining plant nutrients. SA is central to resistance against biotrophs, while JA and to a lesser extent ethylene are antagonistic to SA, contributing to the defence against necrotrophs (Shigenaga and Argueso 2016). Quantitative PCR experiments with isochorismate synthase, which leads to SA production, and 13-lipoxygenase, leading to JA production, showed low or similar expression relative to susceptible controls (data not shown). The supressed JA pathway gene, HORVU7Hr1G041260, is annotated as a ZIM-domain (JAZ) JA repressor, which regulates host and non-host production of reactive oxygen species in the chloroplast and cell death (Ishiga et al. 2013). These responses were in tomato and *Nicotiana benthamiana* against *Pseudomonas syringae*, a necrotroph, and might, therefore, be regarded as antagonistic to biotrophic resistance. Equally, given the involvement of JAZ proteins in the ethylene, salicylic acid, gibberellic acid and auxin plant hormone pathways (Kazan and Manners 2012; Wager and Browse 2012), a barley homologue in the context of *RBgh2* resistance may contribute to an effective immune response. Further indirect evidence of some involvement of the JA pathway was evident in the constitutive suppression of two acyl-coenzyme A oxidases. In *Arabidopsis*, several isozymes are involved in JA synthesis. However, their relevance to barley powdery mildew resistance requires further investigation as they individually appear to influence specific downstream pathogen and cell development functions (Schilmiller et al. 2007). An intriguing gene suppressed in *RBgh2* lines was a WD40 repeat-containing protein (HORVU7Hr1G006140). The orthologue in wheat, *TaHOS15*, increases susceptibility to *Bgt* via a transcriptional repressor complex acting on the chromatin of wheat defence-related genes (Liu et al. 2019), with lower expression fine-tuning innate immunity.

### Concerted gene expression at the 7H *RBgh2* locus

Significant differential gene expression was identified for several resistance genes at the 7H *RBgh2* locus in *Bgh*-inoculated DH APR lines. The majority of these were constitutively expressed. Two are serine–threonine receptor-like kinases; ABC1037 (HORVU7Hr1G001600) (Brueggeman et al. 2006) and *Rpg1* (HORVU7Hr1G001450) a stem rust resistance gene (Brueggeman et al. 2002). Although not reported for the *Rpg1* locus, in wheat partial or slow rusting resistance is associated with partial resistance to mildew. The *Lr34/Yr18/Pm38* gene encodes an ABC transporter (Krattinger et al. 2009) while the gene or tightly linked genes providing *Lr67/Yr46/Sr55/Pm46* resistances is yet to be isolated (Herrera-Foessel et al. 2014).

Two NB-ARC LRR disease resistance-like genes (HORVU7Hr1G002350 and HORVU7Hr1G001540) and kinase-like domain-containing proteins (HORVU7Hr1G001490 and HORVU7Hr1G001600), together with predicted gene models, were also constitutively expressed at the 7H *RBgh2* locus and may have a role in mildew resistance. Kinases are sometimes required and co-expressed with NBS-LRR genes to provide disease resistance (Ade et al. 2007). One of the predicted genes, HORVU7Hr1G000050, had no annotated protein domain family; however, a PANTHER classifier (PTHR23155:SF1087) describes the protein as a putative disease resistance protein and is classed as an antimicrobial response protein. A second predicted gene model, HORVU7Hr1G001520, lacked annotation (Table 2). The range of resistance-associated genes co-expressed at the *RBgh2* locus suggests a pathogen other than barley powdery mildew may equally have initiated directional selection.

### The 7H *RBgh2* locus contains a novel *Mlo*-like ancient paralogue

HORVU7Hr1G002390, which lies within the *RBgh2* locus, lacked expression in the APR DH lines and PCR data indicated substantial disruption of the 3′ end of the gene. HORVU7Hr1G002390 is a paralogue of the prototypic seven-transmembrane domain MLO protein, mutations of which confer *Bgh* race non-specific penetration resistance (Büschges et al. 1997), with 32% amino acid homology and six transmembrane domains. Like *Mlo*, HORVU7Hr1G002390 appears to have a calmodulin (CaM)-binding C-terminus domain (Kim et al. 2002a, 2002b) with at least three conserved hydrophobic residues predicted to be important for binding. CaM is a calcium sensor and messenger intermediate that binds MLO for full susceptibility to *Bgh* (Kim et al. 2002b; Kusch and Panstruga 2017) and is structurally similar to G-protein-coupled receptors although MLO was found to function independently of heterotrimeric G proteins. The cv. Baudin transcript, by contrast, encodes a protein that appeared, by *in silico* protein structural analysis, to bind both G-protein and calmodulin to form a trimeric protein structure (Figure S9). Although further evidence is needed, suppression of G-protein-binding activity in *RBgh2* lines indirectly supports this domain.

The involvement of HORVU7Hr1G002390 in *RBgh2* barley powdery mildew resistance is circumstantial and is qualified by a current lack of gene complementation or silencing. This study is based on comparisons to the barley reference genome (cv. Morex). Plant genomes show extensive presence/absence and copy number variations, often involving resistance gene homologues (Jayakodi et al. 2020). Such differences may have affected the expression analysis, while the existence of other genes conferring *RBgh2* resistance in the parental landrace Eth069 cannot be discounted. The nature of HORVU7Hr1G002390 is further complicated by the cv. Baudin transcript containing an alternative ATG start codon in the first exon (Figure S8), which we assume does not affect function. Despite this, the recessive nature of the resistance and suppression of G-protein-binding activity are suggestive of HORVU7Hr1G002390 involvement. G-protein subunits are evolutionarily conserved, and in mammals, cAMP levels following G-protein ligand binding are a key regulator of innate immune responses, transcription and translation (Ho et al. 2009; Serezani et al. 2008; Ye et al. 1996). Research in barley is lacking but in *Arabidopsis*, the Gα subunit was recently shown to mediate innate immunity against bacterial flagellin (Xu et al. 2019).

*RBgh2* resistance is recessive with conspicuous constitutive expression of genes at the *RBgh2* locus. Genome-wide, many of the gene expression changes are associated with innate immunity in the literature (activation of the tryptophan pathway, induction of calmodulin-binding genes and cytochrome P450 monooxygenases, transcription factors and *PR5* and *PR7*) or are a generic response, such as activation of a large number of resistance gene homologues. Constitutive expression changes included suppression of a ZIM-domain (JAZ) JA repressor and a WD40 repeat-containing protein. Together with the macroscopic and cytological observations of Ge et al. (2021), the results in this study indicate a unique mechanism underlying *RBgh2* barley powdery mildew resistance.

## Supplementary Information

Below is the link to the electronic supplementary material.

Supplementary file1 (DOCX 1493 KB)

Supplementary file2 (XLSX 11 KB)

Supplementary file3 (XLSX 887 KB)

Supplementary file4 (XLSX 24 KB)

## Data Availability

The RNA sequence read data in this manuscript have been deposited in the European Nucleotide Archive under BioProject PRJEB46882, accession numbers ERR6473254-ERR6473324. Gene expression analyses are available as R-markdown notebooks at https://github.com/ccdmb/barley-DH-pow-rnaseq.
