# Supplementary material for: A unique resistance mechanism is associated with RBgh2 barley powdery mildew adult plant resistance
Source: Theor Appl Genet. 2023 May 30;136(6):145. doi: 10.1007/s00122-023-04392-0 (PMC10229466; doi:10.1007/s00122-023-04392-0)
Supplement: Supplementary file 1 — Supplementary file1 (DOCX 1493 KB) [file 122_2023_4392_MOESM1_ESM.docx]

Supplemental Figures

**A unique resistance mechanism is associated with *RBgh2* barley powdery mildew adult plant resistance**

Paula Moolhuijzen, Cynthia Ge, Elzette Wentzel, and Simon R. Ellwood*

Centre for Crop and Disease Management, School of Molecular and Life Sciences, Curtin University, Bentley, WA 6102, Australia

*Corresponding author: [srellwood@gmail.com](mailto:srellwood@gmail.com)

**Supplementary Figure S1**. Total barley RNA sequence reads per Baudin x Eth069 doubled haploid (DH) sample mapped to the IBSC cv Morex reference genome v2, based on four replicates for each of four adult plant resistant (APR) and susceptible lines. Susceptible genotypes are shown in red and APR genotypes in blue. Samples inoculated with *Bgh* are indicated in yellow and non-inoculated in green.

**Supplementary Figure S2**. Contributions of the first two principal components of gene expression for all Baudin x Eth069 doubled haploid (DH) lines and their replicate samples. *Bgh*-inoculated samples with either *RBgh2* barley powdery mildew adult plant resistance (APR) or susceptibility (S) are shown in green and purple, respectively. Non-inoculated control samples are shown in red and in blue, respectively.

**Supplementary Figure S3**. Principal component scores of SDEGs from barley Baudin x Eth069 doubled haploid samples showing the effect of treatment and genotype for SDEGs. *Bgh*-inoculated samples for DH lines with *RBgh2* barley powdery mildew adult plant resistance (APR:Inoculated) and susceptibility (S:Inoculated), are shown in green and purple, respectively. Non-inoculated controls for DH lines with *RBgh2* APR (APR:Control) and susceptibility (S:Control) are shown red and in blue, respectively.

**Supplementary Figure S4**. Tryptophan pathway gene expression in adult plant resistant (APR) and susceptible (S) DH genotypes for *Bgh*-inoculated and non-inoculated controls. Key enzymes are indoleglycerol phosphate aldolase (*trpA*, HORVU7Hr1G075710), tryptophan synthase (*trpB*, HORVU1Hr1G093480 and HORVU7Hr1G092800), indole-3-glycerol phosphate synthase (*trpC*, HORVU7Hr1G114660 and HORVU5Hr1G052150), anthranilate phosphoribosyl transferase (*trpD*, HORVU4Hr1G083210) and anthranilate synthase (*trpG*, HORVU2Hr1G080890 and HORVU4Hr1G061120). The heatmap shows log_2_ gene expression.

**Supplementary Figure S5**. Distribution of genes showing significant differential expression across the barley genome in adult plant resistant (APR) DH lines during powdery mildew infection, compared to susceptible DH lines. Gene counts in a 100 kb window are shown in red and the log_2_ fold change of gene expression in blue. The position of the closest *RBgh2* marker is indicated on chromosome 7H within a cluster of highly expressed genes in APR DH lines.

**Supplementary Figure S6**. Gene expression across all samples are shown for the top 40 most significant differentially expressed genes (log_2_) in *Bgh*-inoculated adult plant resistant (APR) doubled haploid (DH) lines compared to susceptible (S) DH lines.

**Supplementary Figure S7**. Chromosome 7H *Mlo*-like HORVU7Hr1G002390 predicted mRNA and protein sequences. Tracks, from top to bottom, show the 5,745bp reference cv Morex chromosome 7H region (4,607,380-4,613,125bp), susceptible DH line read coverage, splice junctions and alignments, transcript (mRNA) and predicted protein, and cv Morex reference genome v2 HORVU7Hr1G002390 transcripts.

**Supplementary Figure S8**. Protein alignment of the *Mlo*-like gene HORVU7Hr1G002390, a cv Baudin transcript (tr_12967), and the chromosome 4H *Mlo* gene (HORVU4Hr1G082710). Boxed in red is the predicted barley calmodulin (CaM) binding region. Hydrophobic residues predicted to be important for CaM binding are shown as conserved (*) and not conserved (-).

**Supplementary Figure S9**. Predicted conformation of the MLO-like HORVU7Hr1G002390 protein binding to calmodulin (HORVU2Hr1G016740) and G-protein (HORVU7Hr1G008720.4) in AlphaFold2 (Jumper et al. 2021). The MLO-like protein is based on cv Baudin transcript tr_12967 protein translation. The figure on the left shows transmembrane beta-sheets in green with docking of calmodulin (purple) to the binding motif (pink). G-Protein (brown) docking is predicted at the surface of the transmembrane terminus. Surface topography of the complex is shown on the right-hand side.
